# P009 Methotrexate Osteopathy: A Missed Diagnosis?

**DOI:** 10.1093/rap/rkag113.039

**Published:** 2026-11-03

**Authors:** Anna Jiang, Christine Fox

**Affiliations:** University Hospital Dorset, Bournemouth, Christchurch and Poole, United Kingdom; University Hospital Dorset, Bournemouth, Christchurch and Poole, United Kingdom

## Abstract

**Introduction:**

Methotrexate (MTX), a disease-modifying anti-rheumatic drug (DMARD), has been used as a first-line therapy for multiple rheumatic conditions, including rheumatoid arthritis (RA) (1). MTX is a highly effective alternative to steroids, with fewer side effects that support longer-term patient adherence (2). For many years, the side effect profile has been hotly debated. Recently, the rheumatology community has collectively eased its use, though it is still prescribed with caution. MTX has been positively associated with reduced inflammation-driven bone loss. However, long-term MTX use raises concerns about bone tissue health. Rarely, methotrexate-induced osteopathy is encountered by unsuspecting clinicians (3).

**Case description:**

Patient A was diagnosed with Rheumatoid arthritis in 2019 after experiencing pain in her left foot, both hands, right knee, and neck stiffness. Her rheumatoid factor was 38, and she was diagnosed with seropositive rheumatoid arthritis. Her initial X-ray showed preserved joint spacing and good bone density in her hands. She was put on methotrexate 10mg and folic acid 5mg weekly. Her disease was well controlled until 2024, when she experienced right ankle swelling, needing steroid injections, and increasing methotrexate to 15mg weekly. She experienced hallucinations secondary to the steroids; thus, methotrexate was instead increased to 20mg weekly. In October 2024, she presented to A+E after a trip and fall at work, fracturing her left medial tibia. At her 2025 orthopaedic follow-up, she reported poor weight bearing for weeks after she jolted her knee while opening a heavy (but empty) drawer at work. MRI confirmed another right medial femoral condyle fracture, and conservative management was recommended. In June 2025, sulfasalazine 1g twice daily and weekly methotrexate (increased to 25mg) were added for ongoing right foot pain and ultrasound-confirmed knee inflammation. Later in August, she was still struggling to walk (DAS 28 of 3.98 and a CRP of 1), and she was started on Adalimumab 40mg every 2 weeks. Despite all these new medication trials, she still experienced ongoing pain in her foot and knee (CRP of 3.38). An MRI scan of her lower limbs was performed and identified multiple stress fractures of her right and left foot, and right knee. Her DEXA scan showed osteoporosis of the L1-4 vertebras and the left femoral neck. Her methotrexate was stopped, and she was started on teriparatide injections with a plan for 24 months of treatment followed by zoledronate infusions.

**Discussion:**

MTX-induced osteopathy is a rare but increasingly recognised complication of long-term low-dose MTX. The pathophysiology remains unclear, but data suggest MTX reduces osteoblast activation and differentiation, impairing bone formation. In vitro, it increases pro-inflammatory cytokines, correlating with enhanced osteoclastogenesis and impaired osteocyte mechanotransduction in humans(4). In rats, MTX promoted osteocyte apoptosis and osteoclast proliferation(5). Osteocytesare responsible for detecting microcracks and mechanical strain in bone, thus mediate the subsequent repair and remodelling responses(4). By limiting osteocyte numbers, MTX impairs the bone’s ability to recognise and repair microdamage, predisposing it to stress fractures. Given the rarity of MTX-induced osteopathy, our understanding is limited; the long-term effects on bone regulation after discontinuation remain unclear, and ongoing clinical awareness is needed.

Symptoms are described as a triad of pain, osteoporosis, and stress fractures of atypical locations(6,7). Our case confirms the literature impression that it is often misdiagnosed as arthritis, leading to the continuation/escalation of MTX. A typical presentation of MTX-induced osteopathy in a woman of 60-70 with longstanding MTX use (on average six years) on a background of RA (8). It commonly presents as stress fractures in the distal and proximal tibia, calcaneus, and metaphyseal regions, often bilateral and multiple (8). The extent and atypical appearance of these fractures which ultimately made the diagnosis in this case. Most of these patients meet the criteria for osteoporosis, but few have typical osteoporotic fractures (e.g., vertebral) (9). Imaging modalities used to aid diagnosis are preferably MRI rather than X-ray, as the fractures are often transverse and nondisplaced (7). The most important initial step is discontinuing MTX, which allows most patients to achieve complete fracture healing, whereas continuation increases recurrence risk and may be harmful (9). Commencing osteo-forming treatment at diagnosis is advised to maximise bone healing, as in this case (8).

**Key learning points:**

• MTX-induced osteopathy is a rare complication affecting patients with chronic disease. Its recognition is often limited and likely under-diagnosed. Early diagnosis leads toearly treatment.

• Typical signs include long-term MTX use; recurrent atypical fractures of the tibia, calcaneus, or foot; with osteoporosis on DEXA scan and the absence of typical osteoporotic fractures. MRI is often needed to aid diagnosis.

• Stopping MTX is essential to promote bone healing and prevent recurrent fractures. Osteo-forming drugs like Teriparatide can help to accelerate the bone healing process.

